# A Case of Feline Leishmaniosis with Panniculitis

**DOI:** 10.1155/2023/8864790

**Published:** 2023-01-11

**Authors:** Dimitrios Matralis, Ioanna Papadogiannaki, Evi Gkerdidani, Eleni Patsoula, Nikolaos Tegos, Emmanouil Papadogiannakis

**Affiliations:** ^1^Vets 4 life, Small Animal Clinic, Pikermi, Athens, Greece; ^2^Laboratory for the Surveillance of Infectious Diseases, School of Public Health, University of West Attica, Athens, Greece

## Abstract

Leishmaniases are a group of diseases caused by protozoa of the genus *Leishmania* and transmitted mainly by the bite of sand fly vectors. Cats are infected with at least 6 species of *Leishmania*. Significant associations have been found between feline leishmaniosis and coinfections mainly with FIV and/or FeLV. A 7-year-old castrated male, domestic short-haired cat was presented with unknown history and cutaneous and ocular lesions. A raised, semifirm swelling on the forehead was observed along with periocular hypotrichosis and conjunctival and third eyelid edema. The indications for pursuing a diagnosis of leishmaniosis are variable, and differing presentations may require the use of different tests. Diagnosis of feline leishmaniosis with panniculitis caused by *Leishmania infantum* was made by cytology, histopathology, and PCR and *Leishmania* antibodies (IFA). The cat responded to therapy with meglumine antimoniate and allopurinol.

## 1. Introduction

Feline leishmaniosis (FeL) is a vector-borne disease with *Leishmania infantum* being the species more frequently reported in the European countries [1]. Although FeL is a systemic disease, ulcerative and nodular skin lesions are the predominant cutaneous signs [1, 2]. Diagnosis of FeL is made by cytology, histopathology, serology, and polymerase chain reaction. This report describes a case of FeL with panniculitis, a cutaneous lesion reported for the first time in this feline disease.

## 2. Case Description

A 7-year-old castrated male, domestic short-haired cat from the greater Athens area, Greece, was admitted to a veterinary clinic with facial swelling and eye lesions. The cat was strayed and presented for examination with unknown history.

**Figure 1:**
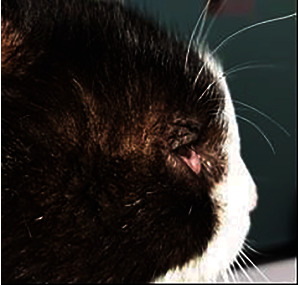
Swelling on the forehead is obvious along with periocular hypotrichosis.

A raised, nonalopecic, semifirm, poorly demarcated, painless swelling of the forehead was observed. Periocular hypotrichosis, edema, and slight hyperpigmentation were additional skin lesions (Figure 1).

**Figure 2:**
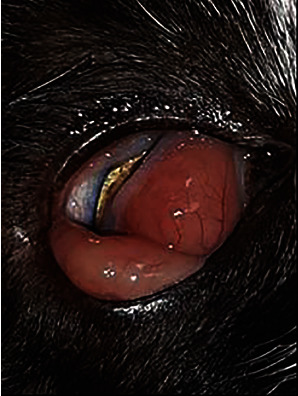
Edema of the conjunctiva and third eyelid of the right eye.

Apart from skin lesions, edema of the conjunctiva and third eyelid of the right eye were also observed (Figure 2). Clinical examination revealed no other abnormalities.

Differential diagnosis included subcutaneous abscess, atypical bacterial and mycobacterial infection, deep fungal infection, and neoplasia.

A blood sample for complete blood count and biochemistry profile was obtained, as well as fine needle aspirates (FNA) and trucut biopsy from the swelling of the forehead for cytological and histopathological examination. The patient had a nonregenerative anemia, hyperglobulinemia, and thrombocytopenia. Further suggested serological tests for vector-borne diseases (e.g., mycoplasmosis and anaplasmosis) were not done due to financial restrictions. Hematological and biochemical abnormalities are shown in Table 1.

Urinalysis showed no abnormalities.

**Figure 3:**
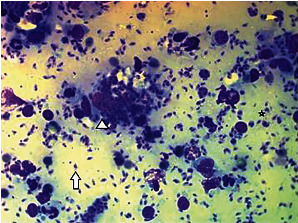
Photomicrography of the swelling of the forehead of the cat with leishmaniosis showing granulomatous inflammation with high number of *Leishmania* amastigotes, both intracellularly (arrowhead) and extracellularly (arrow). In some amastigotes, the basophilic rod-shaped kinetoplast is clearly visible (asterisk) (Diff-Quick stain, x400).

Cytology revealed a granulomatous inflammatory reaction with high number of basophilic structures compatible with *Leishmania* amastigotes, both intracellularly and extracellularly (Figure 3).

**Figure 4:**
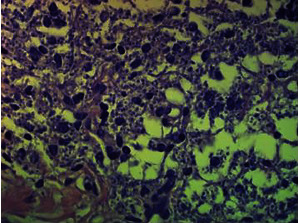
Photomicrography showing granulomatous panniculitis with high number of *Leishmania* amastigotes (Hematoxylin and Eosin stain, x400).

Histopathology revealed granulomatous panniculitis with high number of microorganisms with morphology compatible with *Leishmania* amastigotes (Figure 4).

Serology for *Leishmania* antibodies (IFA test) [3] was positive (titer 1/800).

**Figure 5:**
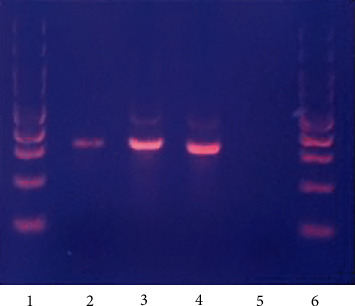
PCR amplified products (lanes 1 and 6: 100 bp DNA ladder; lane 2: FNAB sample; lane 3: positive control for *L. infantum*; lane 4: ATCC NR-50127 positive control for *L. tropica*; lane 5: negative control).

For identification of the *Leishmania* species involved, extraction of genomic DNA from the obtained FNA material was performed, using a robotic DNA extraction protocol, followed by PCR of the ITS1 region (LITSR and L5.8S primers) [4]. The amplified PCR product corresponded to the expected 300-350 bp for *Leishmania* spp. (Figure 5). Sequencing analysis was then carried out using the abovementioned primers. The obtained sequence was assessed in BLAST (Basic Local Alignment Search Tool, available at https://blast.ncbi.nlm.nih.gov/Blast.cgi) for similarities, confirming the identity of the sample as *Leishmania infantum*, as it presented with 99,6% similarity with GenBank deposited sequences, regarding *Leishmania infantum* isolates (internal transcribed spacer 1 and 5.8S ribosomal RNA gene).

The diagnosis of feline leishmaniosis caused by *L. infantum* was made.

Meglumine antimoniate (50 mg/kg SC q24h for 30 days) and allopurinol (10 mg/kg PO q24h for 5-6 months) were given.

**Figure 6:**
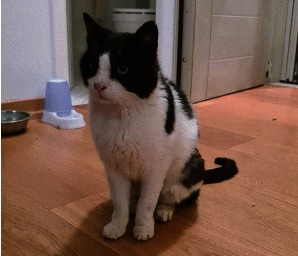
Four months after the start of therapy, the cat looks normal.

Significant clinical improvement was noticed one month after the start of therapy. The owner was advised to continue therapy with allopurinol alone. A second clinical evaluation performed after 4 months revealed no abnormalities (Figure 6). However, several hematological and biochemical abnormalities still remain, as shown in Table 2.

## 3. Discussion

Although the dog is considered the main reservoir of *Leishmania infantum* in endemic areas, such as Greece, an increasing number of clinical cases of FeL have been reported over the last decades, and the cat's role as reservoir host is revalued [3].

Even though skin or mucocutaneous lesions are the most common clinical findings, FeL is a systemic disease with nonspecific signs (depression, malaise, weight loss, or fever) present in about 20-30% of cases and other clinical signs (e.g., respiratory and gastrointestinal) being sporadically findings [1, 3].

Ulcerative dermatitis and nodular lesions are the most frequently reported skin manifestations [1, 2]. Crusty ulcerative often symmetrically distributed lesions with raised margins have been observed on pressure points [3, 5]. Nodules may be single, multiple, or diffuse, firm, alopecic, and nonpainful [6]. They are generally small (<1 cm), mainly distributed on the head and rarely on the limbs or trunk [2, 7]. To the best of the authors' knowledge, panniculitis has not been reported in feline leishmaniosis so far. Accordingly, feline leishmaniosis should be added to the list of differential diagnosis when the cat is presented with cutaneous lesion similar to abscess and/or conjunctival or third eyelid edema mainly in endemic areas of leishmaniosis or in cases of rehomed cats from endemic areas.

Clinicopathological abnormalities more frequently reported in FeL are polyclonal gammopathy and nonregenerative anemia [2]. Similar clinicopathological findings were found in our case, in line with literature. Significant associations have been found between coinfections (e.g., FIV and/or FeLV) and FeL, and it has been estimated that about half of the FeL cases reported in literature were associated with impaired immune competence caused by coinfections or comorbidities [8]. In the present case, FIV and FeLV infections were excluded. Although the cat in our case was not tested for other vector-borne diseases (i.e., ehrlichiosis, anaplasmosis, and bartonellosis), clinical presentation, laboratory abnormalities, and mainly good response to specific therapy for leishmaniosis made these coinfections unlikely.

It has recently been reported that cats from endemic areas produce IFN-*γ* after *ex vivo* blood stimulation with *Leishmania* soluble antigen and therefore are able to activate a cell-mediated adaptive immune response against the parasite [9]. This explains, at least in part, the low number of amastigotes usually found in FeL and the reason why in such cases immunohistochemistry is needed to confirm the diagnosis [3]. However, in our case, we witnessed a high number of amastigotes both in cytology and in histopathology. This probably means that in this case, there might be a suppression of the protective cell-mediated immune response of the cat facilitating the multiplication of the parasite. Alternatively, the cat would be in a transitional period before emergence of systemic FeL, as it has been confirmed the potential progression of *Leishmania* infection to disease in cats even in the absence of comorbidities [10].

Molecular methods, such as sequencing, can be used to identify the *Leishmania* species involved, as it was successfully applied in this case [4].

Treatment of cats with FeL caused by *L. infantum* is empirical and based on off-label use of the more common drugs prescribed to dogs with canine leishmaniosis, such as allopurinol and/or meglumine antimoniate or even miltefosine [1, 8]. Miltefosine carries the risk for haemolytic anemia in the cat [3]. Although the treatment protocol consisted of allopurinol and meglumine antimoniate is off-label in cats, it has been reported quite effective [11]. This later protocol proved also effective in our case, because during and after treatment there were no abnormalities reported by the owner, the cat maintained a normal physical condition, and there was total remission of cutaneous and ocular lesions. However, several laboratory abnormalities still remain, and this is in line with the results of other similar studies [11]. It is possible that a reduced number of viable parasites may persist after treatment. On the other hand, in the cat, cutaneous adverse drug reactions have been observed with allopurinol treatment [10, 12]. Based on the above, cats with FeL should be monitored very carefully for adverse effects during treatment (particularly cats affected by renal disease) and for possible clinical recurrence after stopping the therapy [11].

## 4. Concluded Remarks

Feline leishmaniosis must be considered as one of possible etiological agents when the cat is presented with cutaneous lesions reminiscent an abscess and/or conjunctival or third eye-lid edema, especially in endemic areas of leishmaniosis or in rehomed cats from an endemic area.

## Figures and Tables

**Table 1:** Clinical pathology results before treatment.

Parameter	Initial examination	Normal range
HCT	26,8%	30-45%
HGB	8,9 g/dl	9-15 g/dl
PLTs	108,000/*μ*l	175,000-600,000/*μ*l
Total proteins	9,2 g/dl	5,8-8 g/dl
GLOB	6,2 g/dl	3,8-4,8 g/dl
*γ*-GLOB	30%	7,5-15%
Sodium	162 mmol/l	140-160 mmol/l
*Leishmania* antibodies (IFA)	Positive (1/800)	Negative
FeLV	Negative	Negative
FIV	Negative	Negative

**Table 2:** Clinical pathology results after treatment.

Parameter	4 months after the start of therapy
HCT	27,5%
HGB	9,8 g/dl
PLTs	102,000/*μ*l
Total proteins	6,9 g/dl
GLOB	4,7 g/dl
*γ*-GLOB	21%
Sodium	155 mmol/l
*Leishmania* antibodies (IFA)	Positive 1/200
FeLV	Negative
FIV	Negative

## Data Availability

The data used to support the findings of this study are included within the article.
